# Insights into Physicochemical Characteristics, Flavor Development, and Microbial Succession During the Natural Fermentation of Sichuan-Style Black Soybean Soy Sauce

**DOI:** 10.3390/foods14234049

**Published:** 2025-11-26

**Authors:** Yutian Xie, Shenglan Liao, Youming Li, Xianbin Wang, Yunhao Lu, Qixu Fu, Qiang He, Yuanlong Chi, Zhenghong Xu

**Affiliations:** 1 Innovation Center for Advanced Brewing Science and Technology, College of Biomass Science and Engineering, Sichuan University, Chengdu 610065, China; 2 Yuanjingda Food Co., Ltd., Luzhou 646000, China; 3 Key Laboratory of Monitoring and Assessment on Novel Food Raw Materials, State Administration for Market Regulation, Sichuan University, Chengdu 610065, China; 4 Luzhou Laojiao Group Co., Ltd., Luzhou 646000, China

**Keywords:** Sichuan-style soy sauce, characteristic flavors, microbial succession, correlation analysis, metabolic pathways

## Abstract

Sichuan-style black soybean soy sauce is a traditional fermented condiment renowned for its complex and regionally distinctive flavor profile. This study systematically investigated the physicochemical properties, flavor compounds, and microbial succession during six months of natural fermentation to elucidate the mechanisms underlying its unique flavor formation. Results showed that the amino acid nitrogen level increased to a peak of 1.37 g/100 mL before stabilizing at 1.01 g/100 mL, accompanied by a continuous rise in total acidity (0.69–2.75 g/100 mL). A total of 132 volatile compounds were identified, with esters (e.g., hexanoic acid, methyl ester, hexadecanoic acid, and methyl ester), alcohols (e.g., (E)-2-hepten-1-ol and trans-2-undecen-1-ol), and aldehydes (e.g., benzaldehyde and benzeneacetaldehyde) serving as key differentiating components. Nine taste-active (TAV ≥ 1) and 22 odor-active (ROAV ≥ 1) compounds were recognized as major flavor determinants, among which methional (ROAV = 4.77–119.05), 1-octen-3-ol (ROAV = 40.68–149.35), and 4-ethyl-2-methoxyphenol (ROAV = 4.70–36.26) were dominant contributors imparting sauce-like, mushroom-like, and smoky-clove notes, respectively. Microbial succession revealed a transition from *Weissella* and *Aspergillus* dominance in the early stage to salt-tolerant *Tetragenococcus* and aroma-producing yeasts (*Kodamaea* and *Zygosaccharomyces*) in later phases. Beyond organic acids and fermentation parameters (e.g., pH and salinity), microbial interactions were identified as critical drivers shaping community assembly and succession. Metabolic pathway analysis revealed a stage-dependent mechanism of flavor formation. During the initial stage (0–2 months), *Aspergillus*-mediated proteolysis released free amino acids as key taste precursors. In the later stages (3–6 months), *Tetragenococcus* and aroma-producing yeasts dominated, synthesizing characteristic esters (e.g., benzoic acid and methyl ester, correlated with *Tetragenococcus*; r = 0.71, *p* < 0.05), phenolics (e.g., 4-ethyl-2-methoxyphenol, correlated with *Wickerhamomyces*; r = 0.89, *p* < 0.05), and sulfur-containing compounds (e.g., methional, correlated with *Wickerhamomyces*; r = 0.83, *p* < 0.05).

## 1. Introduction

Soy sauce, a traditional fermented condiment with a history of more than 2500 years, originated in China and represents a profound embodiment of regional culinary culture and fermentation craftsmanship [1]. Traditionally, it is produced through the fermentation of soybeans and roasted wheat with salt, yeast, and lactic acid bacteria, followed by a prolonged period of maturation and enzymatic hydrolysis that imparts its characteristic aroma and flavor [2]. Owing to geographical and cultural diversity, various types of soy sauce have emerged worldwide, including Japanese-style, Chinese-style, Indonesian *Kecap*, and Korean *Kanjang* [3]. In China, soy sauce production exhibits significant regional variations in raw materials, microbial communities, and fermentation conditions, resulting in distinctive flavor profiles. Among these, Sichuan-style soy sauces are particularly renowned for their strong aroma and rich taste, reflecting the region’s warm climate and unique fermentation ecology.

**Figure 1:**
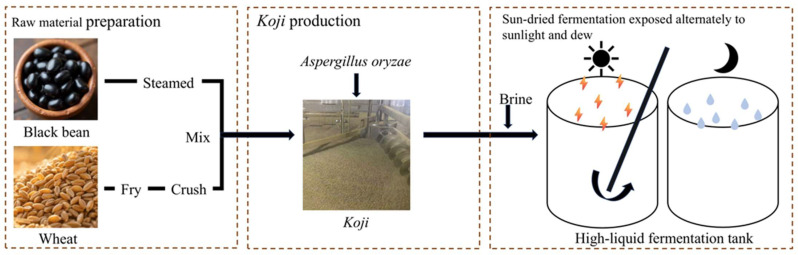
Process diagram for Sichuan-style black soybean soy sauce fermentation.

The uniqueness of Sichuan-style soy sauce largely stems from its traditional sun-dried fermentation process—often described as “sun-dried by day and dew-exposed by night”—a time-honored method that takes advantage of the region’s subtropical monsoon climate to facilitate natural mixed-microbial fermentation. This artisanal process demonstrates how localized environmental factors and traditional practices jointly shape the sensory characteristics of regional soy sauces. Sichuan-style black soybean soy sauce, a representative variant in this category, is a black soybean-based fermented soy sauce characterized by its deep color and complex umami profile derived from diverse microbial activity. The production process typically comprises three major stages: raw material preparation, *koji* production, and sun-dried fermentation exposed alternately to sunlight and dew (Figure 1). The process begins with carefully selected roasted wheat and defatted black soybeans, which are mixed after roasting and steaming to enhance aroma and enzymatic activity. During *koji* making, *Aspergillus oryzae* is inoculated and cultivated under controlled temperature (28–32 °C), humidity, and aeration for approximately 40–44 h, with periodic turning to ensure uniform microbial growth. The mature *koji* is then combined with concentrated brine and transferred into fermentation vats, where long-term fermentation occurs at around 40 °C with regular stirring to promote biochemical transformations. After several months of natural aging, the mash is pressed to obtain raw soy sauce, which is subsequently blended, adjusted, and clarified through sedimentation [4]. *Koji* production, fermentation, and sterilization are considered critical control points that directly influence the final product’s color, aroma, taste, and safety [5].

Previous studies have primarily focused on the compositional and sensory characteristics of soy sauce, particularly the variation in volatile organic compounds and taste-active substances such as free amino acids [6,7]. Comparative analyses have demonstrated that production parameters strongly affect volatile composition. Japanese-style soy sauces generally contain higher levels of alcohols, esters, and furanones that contribute to sweet and floral notes, whereas Chinese-style products—especially those produced by low-salt solid-state fermentation—contain greater concentrations of aldehydes, carboxylic acids, and pyrazines, which impart roasted and savory characteristics [6,7]. The formation of these aroma-active compounds is largely governed by enzymatic reactions mediated by diverse microbial communities during fermentation [8]. However, most previous research has concentrated on Japanese and Cantonese soy sauces [9], and the mechanisms through which microbial communities interact with physicochemical factors to shape the distinctive flavor of Sichuan-style black soybean soy sauce remain insufficiently elucidated.

Recent progress in flavoromics and high-throughput sequencing technologies has provided new perspectives for investigating the interplay between microbial succession and flavor development during soy sauce fermentation. Flavoromics analysis has revealed that amino acids and their derivatives are among the most dynamic flavor precursors, undergoing complex biochemical transformations that generate a wide range of volatile and nonvolatile flavor compounds. Concurrently, microbial diversity analysis based on next-generation sequencing has identified lactic acid bacteria and yeasts as the dominant functional groups contributing to flavor development through amino acid catabolism, ester formation, and other enzymatic reactions [8,10,11].

Therefore, this study aims to systematically elucidate the flavor formation mechanisms of Sichuan-style black soybean soy sauce by integrating flavoromics with microbial diversity analysis to characterize the dynamic succession of microbial communities during fermentation and their associations with key flavor components. Particular attention is given to identifying core functional microorganisms, monitoring changes in aroma-active substances, organic acids, and amino acids, and revealing the correlations between microbial composition and flavor evolution. The findings are expected to provide theoretical insights for optimizing traditional fermentation processes and improving the quality and regional characteristics of Sichuan-style soy sauce.

## 2. Materials and Methods

### 2.1. Soy Sauce Samples Collection

These soy sauce samples were taken from a well-known soy sauce manufacturer in Xichang City, Sichuan Province, China. To ensure biological replication, samples were collected from three parallel high-liquid fermentation tanks. These were periodically sampled at 0, 1, 2, 3, 4, 5, and 6 months of fermentation (designated as 0 M, 1 M, 2 M, 3 M, 4 M, 5 M, and 6 M, respectively). From each tank, samples were gathered from five distinct locations and subsequently pooled to form a composite sample. All samples were immediately transported to the laboratory on dry ice and divided into two aliquots. One aliquot was stored at −80 °C for subsequent microbial analysis, while the other was stored at −20 °C for physicochemical and flavor compound analysis.

### 2.2. Physicochemical Characteristic Analysis

The pH was measured using a pH meter (PHS-3C, Shanghai Yidian Scientific Instrument Co., Ltd., Shanghai, China). Salinity was determined using a salinity meter (SA-287, Guangzhou Mingrui Electronic Technology Co., Ltd., Guangzhou, China). Total acidity was determined by titrating the sample with a 0.05 mol/L sodium hydroxide solution to an endpoint of pH 8.2, and the volume of NaOH consumed was noted [12]. Subsequently, 4 mL of formaldehyde solution was added, and titration continued with 0.05 mol/L NaOH to a final pH of 9.2. The volume of NaOH consumed in this second step was accurately recorded for the calculation of the amino acid nitrogen (AN) content. The reducing sugar content was determined using the 3,5-dinitrosalicylic acid (DNS) method, with a standard curve prepared from glucose standard solutions [10].

### 2.3. Organic Acid and Free Amino Acids

Organic acids and free amino acids were both measured by high-performance liquid chromatography (HPLC, Agilent 1200 Infinity, Santa Clara, CA, USA) on SinoChrom ODS-BP (250 × 4.6 mm, 5 μm) [13]. The diluted samples were sonicated in an ice bath for 10 min, centrifuged (HC-3018R, Anhui Zhongke Zhongjia Scientific Instrument Co., Ltd., Hefei, China) at 4 °C and 8,000 g for another 10 min, and the supernatant was collected for subsequent assays. Proteins in the supernatant were precipitated using 0.4 mol/L perchloric acid and filtered through a 0.22 μm filter for organic acid determination. Free amino acids were treated with a pre-column derivatization reagent (acetonitrile: triethylamine: phenyl isothiocyanate = 88:11:1, *v/v/v*) before HPLC measurement. Appropriate amounts of compound standards were weighed and serially diluted to prepare standard solutions of organic acids (0–2 mg/mL) and amino acids (0–1 mmol/L). Standard curves (“concentration-to-peak area”) were constructed by analyzing solutions at appropriate concentration levels (Table S1). The contribution of individual organic acids and free amino acids to the overall taste was evaluated using taste activity value (TAV).

### 2.4. Volatile Compounds

The volatile flavor compounds were characterized using gas chromatography–mass spectrometry (gas chromatography–mass spectrometry (GC–MS), Shimadzu GCMS-TQ8040NX, Kyoto, Japan), equipped with a DB-5MS capillary column (30 m × 0.25 mm × 0.25 μm, J&W Scientific, Folsom, CA, USA). Precisely measured moromi samples were transferred to a 20 mL solid-phase microextraction vial containing a pre-dried 8 cm diameter rotor and saturated with 1 g NaCl. Simultaneously, 50 μL of 2-octanol was added as an internal standard. The headspace solid-phase microextraction (HS–SPME) method was performed as follows: after equilibration at 60 °C for 30 min, the sample was extracted using a 50/30 μm (DVB/CAR/PDMS) fiber for 30 min under continuous heating and stirring. Following extraction, the fiber was inserted into the gas chromatograph injector for 5 min to desorb the analytes. The initial column temperature was set at 40 °C for 3 min, then increased at 4 °C/min to 105 °C and held for 1 min, further raised at 5 °C/min to 150 °C and maintained for 1 min, and finally increased at 10 °C/min to 250 °C and held for 5 min. The injection port temperature was 250 °C, with no split injection. Triplicate extractions were performed for each sample. Identification of the volatile compounds was achieved by comparing their mass spectra against reference spectra in the NIST20 database and their retention indices (RIs) against literature values, with the RIs being calibrated experimentally using a C_7_–C_40_ n-alkane series. Quantitative analysis was performed using an internal standard (2-octanol, 4 μg/mL), and relative odor activity values (ROAV) were calculated [14].

### 2.5. High-Throughput Sequencing Analysis

The sequencing of microbial communities was carried out by Shanghai Majorbio Bio-pharm Technology Co., Ltd. (Shanghai, China). Total microbial genomic DNA was extracted from soy sauce samples using the E.Z.N.A.^®^ soil DNA Kit (Omega Bio-tek, Norcross, GA, USA) according to the manufacturer’s instructions. The quality and concentration of DNA were determined by 1.0% agarose gel electrophoresis and a NanoDrop2000 spectrophotometer (Thermo Scientific, Waltham, MA, USA) and kept at −80 °C prior to further use. The hypervariable region V3–V4 of the bacterial 16S rRNA gene were amplified with primers 338F (5′-ACTCCTACGGGAGGCAGCAG-3′) and 806R (5′-GGACTACHVGGGTWTCTAAT-3′) and ITS1 region of the fungal were amplified with primers ITS1F (5′-CTTGGTCATTTAGAGGAAGTAA-3′) and ITS2R (5′-GCTGCGTTCTTCATCGATGC-3′). The PCR product was extracted from 2% agarose gel and purified using the PCR Clean-Up Kit (YuHua, Shanghai, China) according to the manufacturer’s instructions and quantified using Qubit 4.0 (Thermo Fisher Scientific, Waltham, MA, USA). Purified amplicons were pooled in equimolar amounts and paired-end sequenced on an Illumina Nextseq2000 platform (Illumina, San Diego, CA, USA) according to the standard protocols by Majorbio Bio-Pharm Technology Co., Ltd. (Shanghai, China). Finally, the optimized sequence was clustered into operational taxonomic units (OTUs). All subsequent bioinformatics analyses were conducted on the Majorbio Cloud Platform (https://www.majorbio.com/tools, accessed on 20 Ausust 2025).

### 2.6. Metabolic Pathway Analysis

Based on the 16S rRNA sequencing data, we used the Phylogenetic Investigation of Communities by Reconstruction of Unobserved States (PICRUSt2) software (v2.6.2) to predict the gene functions of the microbial community. And then mapped these functions to specific metabolic pathways through the Kyoto encyclopedia of genes and genomes (KEGG) database (https://www.kegg.jp/, accessed on 24 September 2025).

### 2.7. Statistical Analysis

Experimental results were expressed as mean ± standard deviation. Flavoromics data were statistically analyzed using *t*-tests (*p* < 0.05), while microbiome datasets underwent Kruskal–Wallis (K-W) tests. Visualization and graphical analysis were performed using Origin 2024 and GraphPad Prism (v10.6.0). The principal component analysis (PCA) and partial least-squares discrimination analysis (PLS-DA) via SIMCA 14.0 software. Principal coordinate analysis (PCoA), Variance partitioning analysis (VPA), redundancy analysis (RDA) and Spearman correlation analysis were analyzed on the online tool of Majorbio Cloud Platform (https://www.majorbio.com/tools, accessed on 15 August 2025).

## 3. Results and Discussion

### 3.1. Dynamic Changes in Physicochemical Properties and Organic Acids

**Figure 2:**
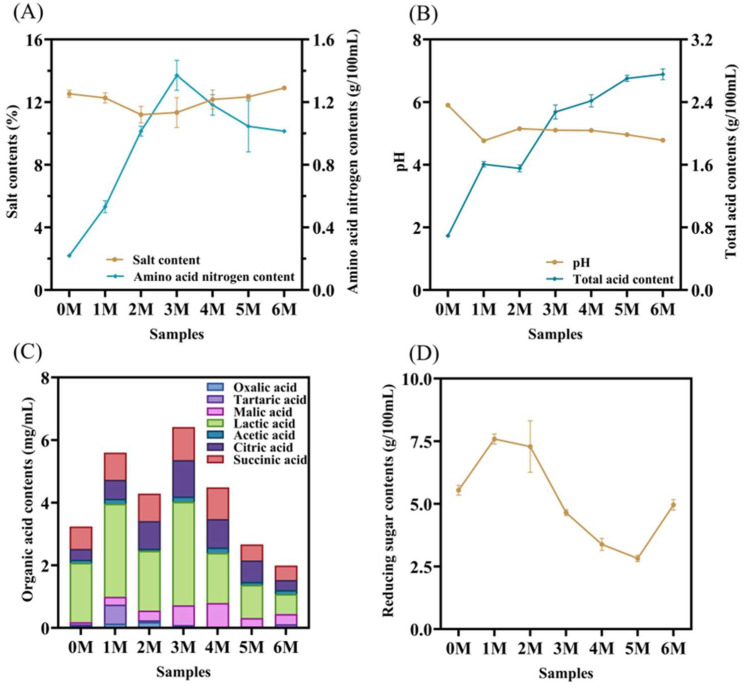
Dynamic changes in physicochemical properties and organic acid contents during the fermentation of Sichuan-style black soybean soy sauce. (**A**) Salt contents and amino acid nitrogen contents. (**B**) pH and total acid contents. (**C**) Organic acid contents. (**D**) Reducing sugar contents.

Physicochemical properties constitute a fundamental material basis for regulating fermentation processes and determining the flavor quality and safety stability of fermented foods [15]. During the fermentation process of soy sauce, the salt content (SA) fluctuated within the range of 11.2% and 12.9% (Figure 2A). The amino acid nitrogen (AN) serves as a key indicator for classifying the quality grade of soy sauce [11]. In the initial fermentation stage, the AN content increased rapidly, reaching a peak of 1.37 g/100 mL by the third month. This rise can likely be attributed to the proteases secreted by *Aspergillus oryzae* and other microorganisms, which hydrolyze proteins in the raw materials into free amino acids and small peptide fragments. Subsequently, the AN content began to decline, falling to 1.01 g/100 mL by the sixth month. This decrease may be associated with the inhibitory effect of increasing salt content on the metabolic activity of *A. oryzae* [16]. Despite this decline, the final AN content still exceeds the threshold specified in the Chinese national standard GB/T 18186–2025 for premium-grade soy sauce [17].

Both pH and total acidity (TA) exhibited notable fluctuations during the first month of fermentation (Figure 2B). Overall, pH displayed a declining trend, whereas TA generally increased, which can be mainly ascribed to the substantial production of organic acids by lactic acid bacteria [18]. The pH and TA present consistent experimental results. An acidic fermentation environment helps suppress the growth of undesirable microorganisms, while an optimal pH promotes yeast proliferation, thereby facilitating the formation of flavor compounds [19].

Organic acids play a crucial role in shaping the flavor profile of fermented foods, not only by providing characteristic sourness and aroma but also by inhibiting the growth of undesirable microorganisms, thus serving as natural preservatives [20]. In this study, seven organic acids were identified during soy sauce fermentation, including lactic acid, succinic acid, and citric acid (Figure 2C). The total content of organic acids initially increased from 3.24 ± 0.11 mg/mL to a peak of 6.41 ± 0.10 mg/mL, followed by a decline to 1.99 ± 0.21 mg/mL by the end of fermentation. This pattern is attributed to the continuous production of organic acids in the early stage, followed by their consumption in esterification reactions during later fermentation [21]. Lactic acid and succinic acid were identified as the predominant organic acids, consistent with previous reports [12]. Lactic acid, which reached a maximum concentration of 3.30 ± 0.09 mg/mL in the sixth month, contributes a mild and lingering mouthfeel to soy sauce, while acetic acid helps balance the overall taste [22]. In fermented foods, lactic acid is primarily produced via the phosphoketolase and glycolysis pathways, representing key metabolic products of lactic acid bacteria [23]. Succinic acid, previously confirmed to impart umami taste [24], increased rapidly from 0.72 ± 0.00 mg/mL to 1.06 ± 0.02 mg/mL by the third month. Both succinic acid and citric acid are central intermediates in the tricarboxylic acid (TCA) cycle [25], which explains their initial increase and subsequent decrease as the fermentation progressed. These organic acids can further react with alcohols and ketones to form aromatic esters such as ethyl acetate [8], thereby enhancing the complexity of the flavor profile. Additionally, the sour and savory characteristics they impart significantly influence the overall sensory quality of soy sauce. The contribution of individual organic acids to the overall taste was evaluated using TAV (Table 1). In the early fermentation stage, the TAVs of lactic acid, acetic acid, citric acid, and succinic acid all exceeded 1, indicating their significant contribution to the sourness of soy sauce. Among all the organic acids with TAV > 1, succinic acid has the highest value and lactic acid has the lowest value. The TAV of all organic acids demonstrated an initial increase, followed by a decrease as ester substances were formed, and this trend persisted until the fifth month. By the sixth month, as organic acids were metabolized, only acetic acid and succinic acid retained TAVs greater than 1, thus preventing excessive acidity in the final product.

Reducing sugars (RS), generated from carbon sources utilized by the fermentation microbiota, act as key precursors in the Maillard reaction. These sugars polymerize with amino compounds in soy sauce to form melanoidins, which are largely responsible for the characteristic reddish-brown color of the product [27]. This reaction also serves as a major pathway for flavor formation. As illustrated in Figure 2D, the RS content increased markedly from 5.54 g/100 mL to 7.59 g/100 mL during the first month of fermentation, likely due to the high enzymatic activity of amylase, protease, glycosidase, and other enzymes produced by *A. oryzae* [11]. Between the second and fifth months, the RS content decreased significantly, which may be attributed to microbial consumption of sugars. It is worth noting that during the fifth to sixth month, the RS content increased again, possibly due to a resurgence in the abundance or activity of *A. oryzae*.

### 3.2. Changes in Free Amino Acids During Fermentation

**Figure 3:**
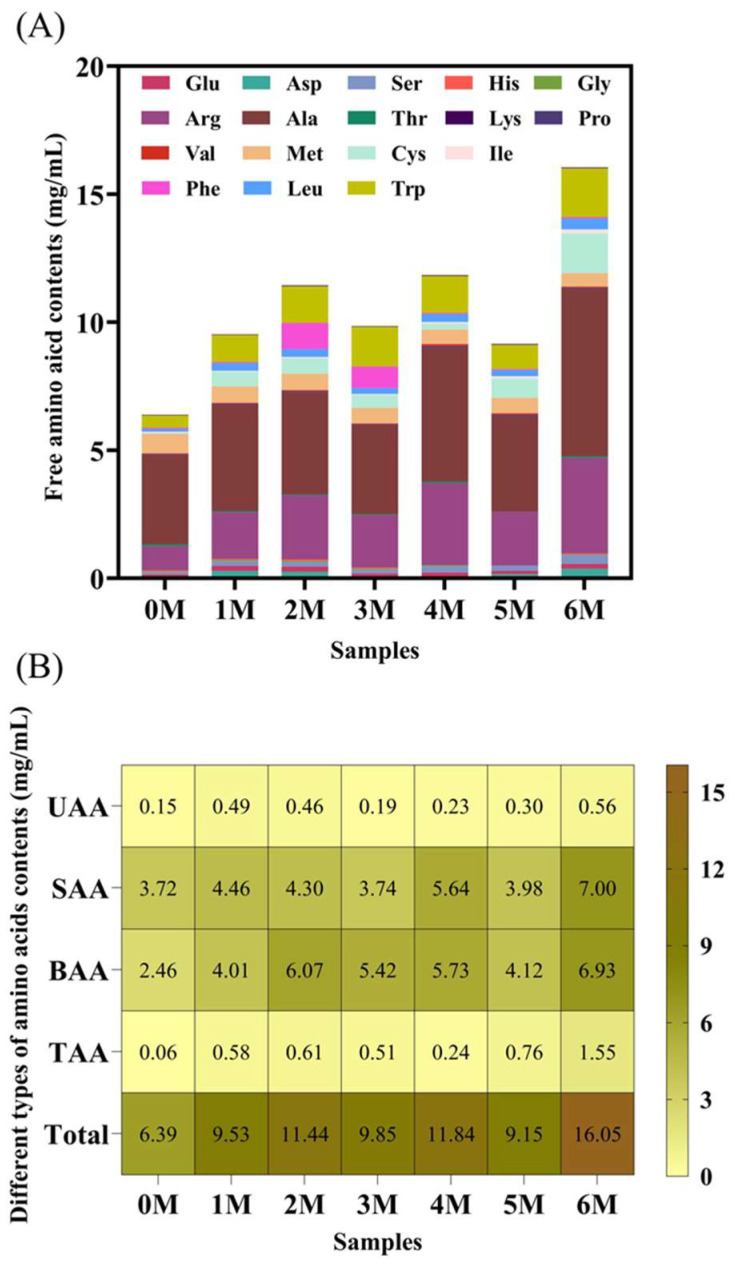
Changes in free amino acids during the fermentation of Sichuan-style black soybean soy sauce. (**A**) Free amino acid contents. (**B**) Different types of amino acid contents. Total: total amino acid contents; UAA: umami amino acid contents; SAA: sweet amino acid contents; BAA: bitter amino acid contents; TAA: tasteless amino acid contents.

Free amino acids serve as critical precursors to volatile flavor compounds and are key indicators of the sensory properties of fermented products [8]. A total of 17 free amino acids were detected during soy sauce fermentation, with the total content increasing from 6.39 ± 0.23 mg/mL to 16.05 ± 3.19 mg/mL as fermentation progressed (Figure 3A). This rise can be attributed to the continuous degradation of proteins in the raw materials [4]. Alanine and arginine were the most abundant amino acids throughout the process. Based on taste characteristics, the free amino acids were categorized into umami, bitter, sweet, and tasteless types [21]. As shown in Figure 3B, sweet and sour-tasting amino acids consistently dominated the profile and were present at significantly higher levels than umami and tasteless amino acids (*p* < 0.05).

TAV was also applied to assess the contribution of individual amino acids to the overall taste (Table 1). Throughout fermentation, only alanine (sweet), arginine and methionine (both bitter) had TAVs greater than 1. Among them, alanine has the highest TAV, while methionine has the lowest. Compared with Month 0, the TAV of arginine and alanine was significantly higher than that of Month 6, while that of methionine was lower than that of Month 6. Soy protein is rich in arginine and is efficiently hydrolyzed by the protease of *A. oryzae*, continuously releasing it. Alanine, which can be generated via the transamination of pyruvate, is also known to accumulate as an intermediate during yeast fermentation [28], underscoring its consistent sensory impact in soy sauce. Methionine is the precursor of the characteristic sulfur-containing flavor compounds in soy sauce [15]. During the fermentation process, it is transformed into other sulfur-containing flavor compounds, becoming the core contributor to the aroma of soy sauce.

### 3.3. Changes in Volatile Flavor Compounds During Fermentation

**Figure 4:**
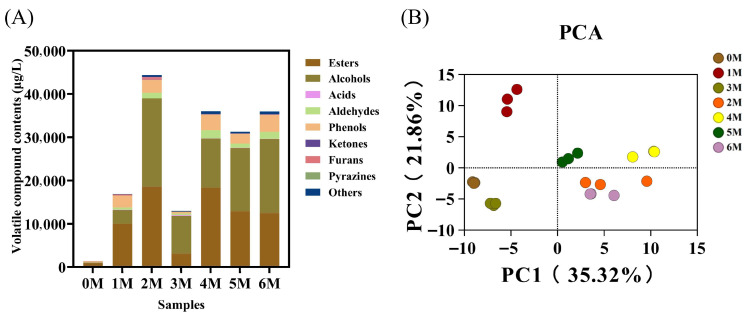
Dynamic changes in volatile flavor compounds during the fermentation of Sichuan-style black soybean soy sauce. (**A**) Content histogram of volatile flavor compounds during fermentation. (**B**) Principal component analysis (PCA).

Flavor represents the most critical quality attribute of soy sauce and is predominantly shaped by the microbial communities within the fermented food ecosystem [12]. A total of 132 volatile flavor compounds were identified by HS–SPME–GC–MS, including 65 esters, 21 alcohols, 11 aldehydes, 8 acids, 6 ketones, 5 phenols, 2 furans, 1 pyrazine, and 13 other compounds (Table S2). Throughout fermentation, the content of volatile compounds in soy sauce demonstrated an initial increase, followed by a decrease, and subsequently stabilized (Figure 4A). Specifically, the content surged from an initial 1414.5 ± 349.85 µg/L to a peak of 43,985.14 ± 10,153.64 µg/L at month 3, before declining and eventually stabilizing at approximately 36,000 µg/L. Consistent with the previous research results [14], the content of volatile flavor substances was relatively low at the mid-fermentation stage (3 M). This may be attributed to the decomposition of early-formed flavor compounds by microorganisms and their subsequent transformation into different flavor substances [29]. Esters (880.16–18,564.99 µg/L), alcohols (146.87–20,406.56 µg/L), and phenols (277.78–3930.99 µg/L) constituted the dominant categories of volatile compounds across fermentation stages, in agreement with previous studies [29], and together form the core aroma profile of soy sauce. Moreover, the composition of these substances also demonstrates the same trend as the total volatile flavor compounds, while the changes in the content of other substances are relatively smaller. These compounds are primarily generated through reactions such as lipid oxidation, protein degradation, the Maillard reaction, microbial metabolism, and alcoholic fermentation [30]. The decrease in alcohol content in the third month might be due to the esterification reaction.

At the initial fermentation stage (0 M), the content of volatile flavor compounds was relatively low. The most abundant compounds at this point were methyl hexadecanoate and 2-methoxy-4-vinylphenol, with concentrations of 468.62 ± 105.11 and 260.04 ± 185.34 µg/L, respectively. During months 1–2, substantial accumulation of esters was observed, including hexadecanoic acid, ethyl ester (7280.86 ± 3933.04 µg/L), and benzoic acid, ethyl ester (2503.76 ± 625.67 µg/L), etc. By the third month, the abundant compounds present in the early stages had been partially depleted, resulting in a somewhat monotonous profile of volatile compounds during the mid-fermentation phase. Through research, the evolution of volatile flavor compounds during soy sauce fermentation has been summarized into two distinct phases: the first phase occurs from the early to mid-fermentation stage, characterized primarily by the decomposition of raw materials; the second phase extends from the mid-fermentation stage through to completion, focusing mainly on the transformation of aromatic substances [30]. In the later stages of fermentation, microbial activity led to the transformation of early-formed flavor compounds into different substances, such as benzeneacetaldehyde and ethyl benzoate, which reached concentrations of 730.82 ± 36.06 and 2259.57 ± 459.22 µg/L, respectively.

However, the relative content of compounds in the sample does not directly reflect their contribution to the overall aroma [31]. Compounds with a relative odor activity value (ROAV) ≥ 1 are considered to contribute significantly to the flavor profile [32]. As summarized in Table 2, 22 volatile flavor compounds were identified as aroma-active substances, including 12 esters, 5 aldehydes, 2 alcohols, 2 phenols, and 1 acid. Among these, Phenol, 4-ethyl-2-methoxy- is a representative aroma compound that imparts a characteristic savory, smoky, and clove-like note central to the typical scent of soy sauce [33]. Its ROAV remained high from the second to the sixth month, increasing from 4.70 to 36.26. Methional, which enhances sauce-like and caramel-like aromas, was also identified as a key aroma compound in Chinese soy sauces [34]. Throughout the fermentation process, its ROAV remains at a high level, rising from 4.77 to 119.05. In addition, 1-octen-3-ol (ROAV = 40.68–149.35, mushroom, earthy, fungal, green, oily, vegetative) and several esters consistently exhibited high ROAV, contributing floral and herbal notes that further enrich the complex bouquet of soy sauce. Furthermore, numerous other esters, including hexanoic acid, ethyl ester (ROAV = 17.52–23.44, fruity, winey, waxy, sweet) and hexadecanoic acid, methyl ester (ROAV = 95.39–1648.23, oily, waxy, fatty, orris), exhibit relatively high ROAVs. Compounds with ROAV ≥ 1 are almost absent in the early fermentation stage. As fermentation proceeded, the number of compounds with ROAV ≥ 1 increased, indicating their growing contribution to the flavor profile.

The content of volatile flavor compounds changed dynamically throughout fermentation, with most compounds accumulating over time and collectively shaping the distinctive aroma profile of soy sauce. To further interpret the GC–MS results, PCA was performed based on the relative content of the compounds (Figure 4B). According to the analysis results, the cumulative variance contribution rate of PC1 (35.32%) and PC2 (21.86%) reached 57.18%. Samples from 0 M, which contained fewer flavor compounds, clustered together with 3 M samples, in which early-formed flavors had been largely transformed. 1 M samples formed a separate cluster, while later-stage fermentation samples also grouped together. PCA revealed distinct clustering patterns among samples from different fermentation stages, confirming clear differentiation among them [15].

**Figure 5:**
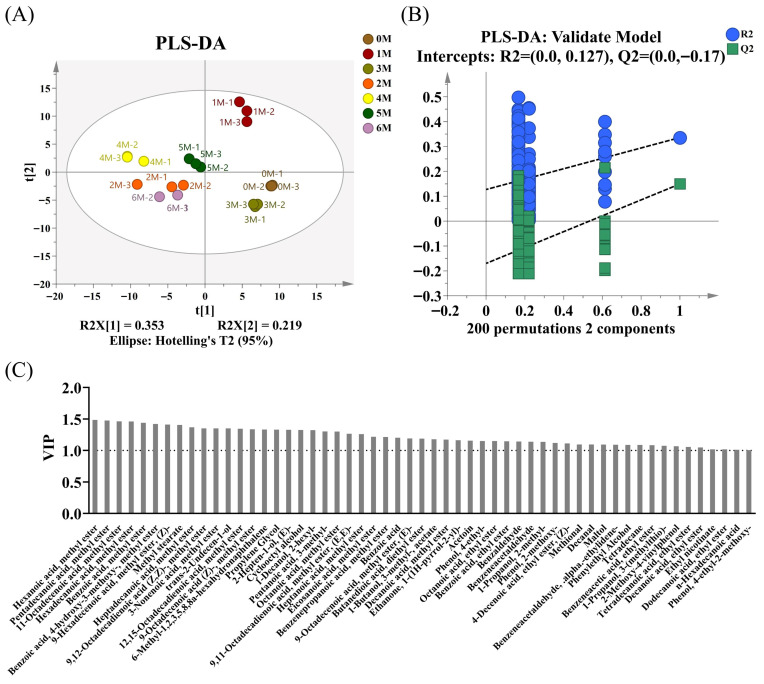
Changes in volatile flavor compounds during different fermentation stages (**A**) Partial least squares discrimination analysis (PLS-DA) score plot. (**B**) Validation plots of the PLS-DA model. (**C**) Variable importance in projection (VIP) plot.

In order to identify the main different aroma substances at different fermentation stages, the PLS-DA model was used for evaluation (Figure 5A). The reliability of the PLS-DA model was validated through 200 permutation tests, yielding R^2^ = 0.127 and Q^2^ = −0.17 (Figure 5B). The regression curves of R^2^ and Q^2^ show an upward trend, and the intercept of Q^2^ is less than 0.05, indicating that the model has passed the permutation test and there is no overfitting phenomenon. As shown in Figure 5C, a total of 55 volatile flavor compounds were identified as differential aroma compounds (VIP > 1, *p* < 0.05), among which 12—including Hexanoic acid, methyl ester (VIP = 1.48, ethereal, fruity, pineapple), Hexadecanoic acid, methyl ester (VIP = 1.46, oily, waxy, fatty), Benzoic acid, methyl ester (VIP = 1.44, floral, fruity), Methyl stearate (VIP = 1.41, oily, waxy) and 9,12-Octadecadienoic acid (Z,Z)-, methyl ester (VIP = 1.35, oily, fatty, woody)—were also characterized as aroma-active compounds. These substances are commonly found in various types of soy sauce and constitute its characteristic aromatic components [29]. However, furanones such as 2,5-dimethyl-4-hydroxy-2H-furan-3-one (HDMF) and 5-ethyl-4-hydroxy-2-methyl-3(2H)-furanone (HEMF) were not detected in the samples, which are considered the characteristic aroma components of soy sauce [35]. They are produced through the thermal degradation of carbohydrates and through microbial metabolism, mainly related to the decarboxylation process of yeast [36]. The lack of such substances may be due to the high-temperature (40 °C) fermentation process in the fermentation tank, which results in insufficient thermal degradation of carbohydrates.

### 3.4. Succession of Microorganisms During Fermentation

**Figure 6:**
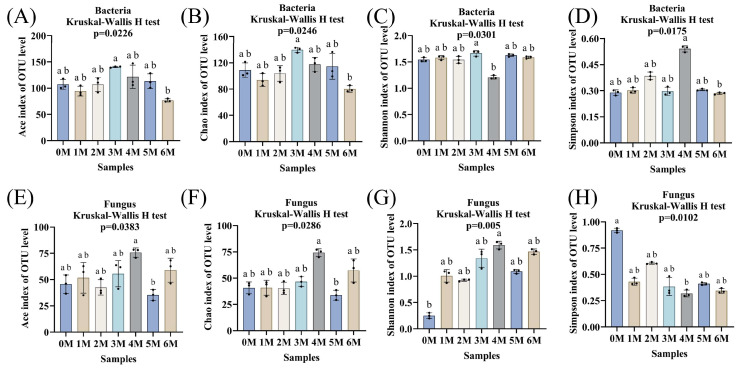
The microbial diversity during the fermentation of Sichuan-style black soybean soy sauce. (**A**,**E**), Ace indices. (**B**,**F**), Chao 1 indices. (**C**,**G**), Shannon indices. (**D**,**H**), Simpson indices. a,b: different letters above the bars denote significant differences (*p* < 0.05).

Through high-throughput sequencing of 16S rRNA gene sequences for bacteria and ITS sequences for fungi from samples collected over the 0- to 6-month fermentation period, a total of 927,318 high-quality bacterial sequences and 861,840 fungal sequences were obtained, which clustered into 1894 bacterial OTUs and 900 fungal OTUs. Alpha diversity indices (Ace, Chao1, Shannon, Simpson) were used to evaluate microbial community richness and evenness (Figure 6A–H). For bacteria, the Ace and Chao1 indices (reflecting richness) and the Simpson index (reflecting diversity) initially increased and subsequently decreased. This pattern may be attributed to the abundance of nutrients in the early stages supporting a wide range of bacterial taxa, followed by intensified competition leading to reduced diversity [14]. The Shannon index remained relatively stable throughout fermentation, except for a noticeable fluctuation in the fourth month. For fungi, the Ace and Chao1 indices remained generally stable, except for significant increases in the fourth month and decreases in the fifth month, suggesting dynamic shifts in fungal richness during mid-fermentation. Both Shannon and Simpson indices indicated that fungal diversity peaked in the fourth month.

**Figure 7:**
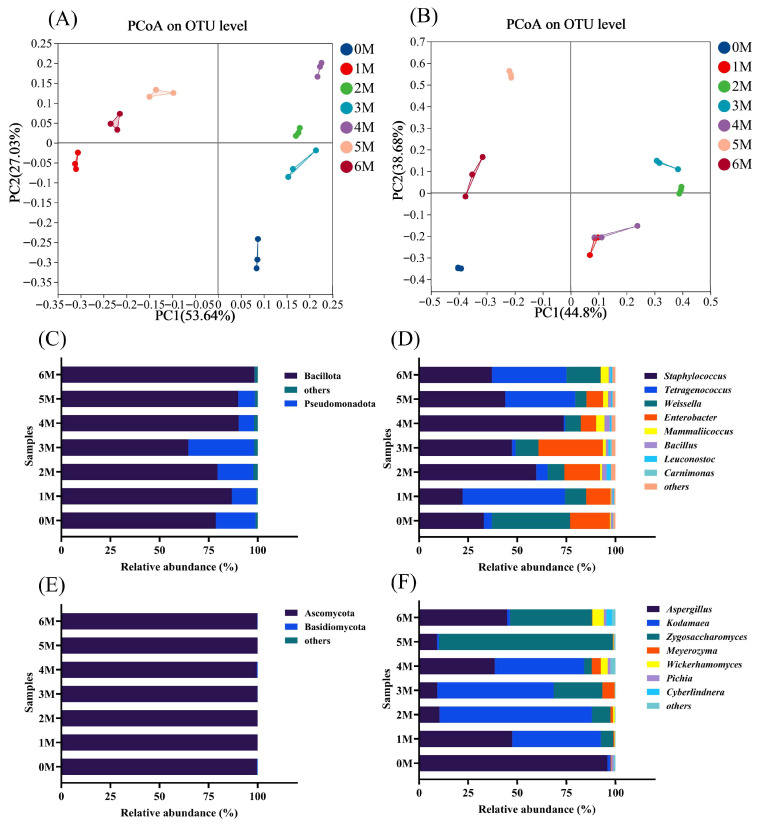
The microbial community composition during the fermentation of Sichuan-style black soybean soy sauce. (**A**,**B**), Principal coordinate analysis (PCoA) of bacteria and fungi. (**C**,**D**), At the phylum level, changes in bacterial and fungal communities. (**E**,**F**), At the genus level, changes in bacterial and fungal communities.

PCoA was further applied to illustrate the microbial diversity across samples [37]. The PCoA revealed clear separation of microbial communities at different fermentation time points (Figure 7A,B), indicating significant temporal succession in both bacterial and fungal compositions. In the bacterial community analysis, both 0 M and 3 M samples were located in the lower-right region, which may explain the relative similarity in volatile flavor compounds between these two stages. Fungal community analysis demonstrated that 1 M and 4 M samples clustered together. For both bacteria and fungi, samples from the early fermentation stages were clearly separated from those of the later stages (5–6 M).

To comprehensively characterize the dynamic changes in the microbial community during fermentation, we analyzed the relative abundances of microorganisms at the phylum and genus levels. At the phylum level for bacteria (Figure 7C), Bacillota (64.65–90.28%) and Pseudomonadota (0.15–33.58%) were predominant. Bacillota increased initially but decreased by the third month, a trend consistent with observations in Cantonese soy sauce [38]. At the genus level (Figure 7D), *Staphylococcus* (22.20–73.68%), *Tetragenococcus* (0.80–52.13%), *Weissella* (5.65–39.91%), and *Enterobacter* (0.15–32.74%) were the dominant genera, consistent with previous reports [39]. *Staphylococcus* maintained a high abundance throughout fermentation, likely due to its tolerance to high salinity and alcohol conditions [16]. *Weissella* was predominant in the initial stage, declined to 5.65–11.59%, and then rebounded to 17.35% by the sixth month. As fermentation progressed, *Tetragenococcus* gradually became dominant in later stages, aligning with earlier findings [40]. This genus can produce organic acids that acidify the fermentation environment, thereby promoting the formation of flavor compounds such as 1-octen-3-ol, 2-methylpropionaldehyde, and phenylethanal [41]. Notably, *Enterobacter*—a potential foodborne pathogen in fermented foods [15]—was present during the first five months but declined markedly by the sixth month.

For fungi, Ascomycota (99.61–99.96%) was the dominant phylum (Figure 7E) and also constitutes the primary component of Cantonese soy sauce [9]. At the genus level (Figure 7F), *Aspergillus* (9.14–95.98%) and *Kodamaea* (0.89–77.60%) were predominant in the early fermentation stage. Although *Aspergillus* is essential during the *koji*-making stage, its growth is inhibited as salinity increases. Meanwhile, *Zygosaccharomyces rouxii* converts amino acids into flavor-active alcohols such as isobutanol, isovaleraldehyde, and 2-phenylethanol, which collectively contribute to the characteristic flavor profile of soy sauce [42]. The abundance of *Zygosaccharomyces* increased as fermentation progressed, peaking in the fifth month (88.69%). This genus is crucial for producing higher alcohols and furans, key aroma compounds in soy sauce [43]. *Kodamaea* remained abundant from the first to the fourth month but declined significantly thereafter. However, its functional role in soy sauce fermentation remains inadequately studied and warrants further investigation [39].

### 3.5. Drivers of Microbial Succession During Soy Sauce Fermentations

**Figure 8:**
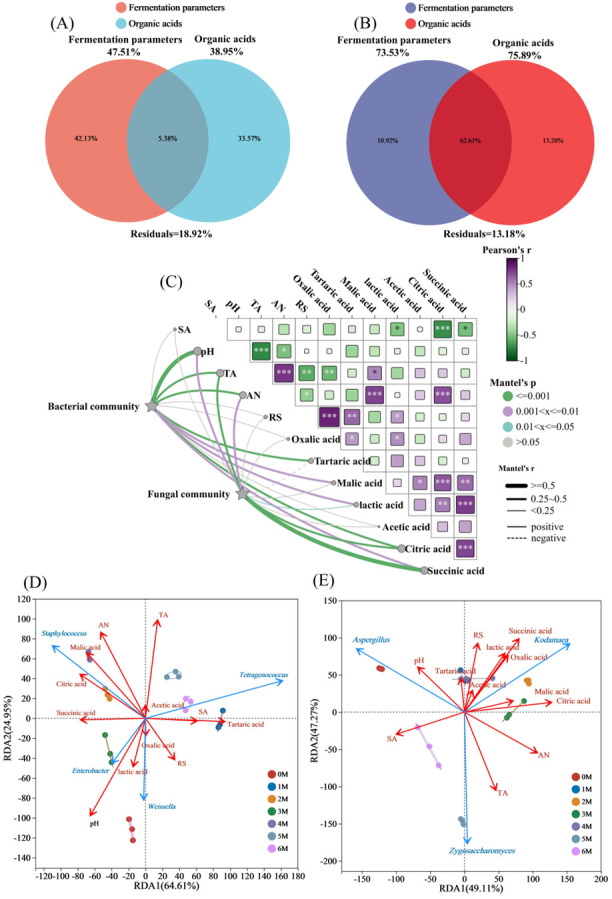
Drivers of microbial succession during the fermentation of Sichuan-style black soybean soy sauce. (**A**,**B**), Analysis of Variance partitioning analysis (VPA) for bacteria and fungi, respectively (SA, Salt contents; AN, amino acid nitrogen contents; TA, total acid contents). (**C**) Mantel test of bacteria and fungi. *** *p* < 0.001; ** *p* < 0.01; * *p* < 0.05. (**D**,**E**), Redundancy analysis (RDA) of bacteria and fungi, respectively.

Microbial succession in fermented foods is governed by complex interactions among physicochemical properties, substrate availability, and metabolic byproducts [15]. Nutrient fluctuations are ubiquitous in fermenting ecosystems [44]. To identify the principal factors driving microbial community assembly in soy sauce, VPA was conducted. Environmental variables were classified into two groups: organic acids and fermentation parameters (pH, salinity, reducing sugars, total acidity, and amino acid nitrogen). The VPA revealed distinct explanatory patterns between bacterial and fungal communities (Figure 8A,B). These factors collectively explained 81.08% of the variance in bacterial communities and 86.82% in fungal communities, leaving 18.92% and 13.18% unexplained, respectively. For bacteria, fermentation parameters contributed most substantially (42.13%), followed by organic acids (33.57%). In contrast, for fungal communities, organic acids exhibited greater explanatory power (13.28%) than fermentation parameters (10.92%). These research results indicate that the fermentation parameters have a significant impact on the composition of the bacterial community in soy sauce. By regulating the fermentation parameters, the structure and function of the bacterial community can be effectively controlled, thereby influencing the quality and flavor of soy sauce. Similarly, modulating the accumulation of organic acids during fermentation can alter the composition of the fungal community.

To further explore the relationships between physicochemical dynamics and microbial succession, Mantel tests were employed to correlate bacterial and fungal community structures with multiple environmental factors [45]. As shown in Figure 8C, pH was significantly correlated with both bacterial (*p* < 0.001, r ≥ 0.5) and fungal (*p* < 0.01, 0.25 < r < 0.5) communities. This can be attributed to the varying pH tolerance among microorganisms: acid-tolerant microbes such as lactic acid bacteria thrive in lower pH environments, while non-acid-tolerant bacteria are inhibited under such conditions [46]. TA was extremely significantly correlated with the composition of bacterial (*p* < 0.001, 0.25 < r < 0.5) and fungal communities (*p* < 0.001, 0.25 < r < 0.5). Salt stress effectively selects for beneficial salt-tolerant microorganisms during fermentation [47]. AN was extremely significantly correlated with bacterial community (*p* < 0.001, 0.25 < r < 0.5) and significantly correlated with fungal community (*p* < 0.01, 0.25 < r < 0.5), as microorganisms require amino acid nitrogen for protein synthesis and other nitrogen-containing compounds to support growth and reproduction. Bacterial community composition was significantly correlated with tartaric acid, malic acid, lactic acid, citric acid and succinic acid (*p* < 0.01, 0.25 < r < 0.5). Fungal community composition was extremely significantly correlated with citric acid (*p* < 0.001, 0.25 < r < 0.5) and succinic acid (*p* < 0.001, r ≥ 0.5).

RDA was performed to clarify key factors influencing microbial community assembly. For bacteria, RDA1 and RDA2 explained 64.61% and 24.95% of the total constrained variance, respectively (Figure 8D). Key physicochemical correlates, including pH (R^2^ = 0.79, *p* = 0.001), TA (R^2^ = 0.56, *p* = 0.001), and AN (R^2^ = 0.57, *p* = 0.002), were strongly correlated with bacterial composition. Among organic acids, tartaric acid (R^2^ = 0.50, *p* = 0.001), malic acid (R^2^ = 0.51, *p* = 0.003), and citric acid (R^2^ = 0.44, *p* = 0.008) were significantly associated with bacterial composition. AN, malic acid, and citric acid were negatively correlated with *Staphylococcus*, the dominant bacterial genus throughout fermentation. For fungi, RDA1 and RDA2 explained 49.11% and 47.27% of the variance, respectively (Figure 8E). SA (R^2^ = 0.47, *p* = 0.007), TA (R^2^ = 0.57, *p* = 0.001), and AN (R^2^ = 0.63, *p* = 0.001) were strongly correlated with fungal composition. Among organic acids, oxalic acid (R^2^ = 0.43, P = 0.006), lactic acid (R^2^ = 0.42, *p* = 0.012), citric acid (R^2^ = 0.73, *p* = 0.001), and succinic acid (R^2^ = 0.70, *p* = 0.001) demonstrated significant correlations. The *Tetragenococcus* is the only bacterium that demonstrates a positive correlation with salinity. This might be due to its superior salt tolerance [48]. As with previous research, the number of *Staphylococcus* was negatively correlated with the salt content of soy sauce, with high salt concentrations inhibiting the growth of inhibiting growth [14]. The formation of reducing sugars is mainly driven by the glycoside hydrolases produced by *Aspergillus* [49]. This also explains why *Aspergillus* is positively correlated with RS. *Weissella* has a positive correlation with lactic acid content. As a type of lactic acid bacteria, it undergoes lactic acid fermentation, secretes various enzymes, promotes the hydrolysis of raw materials, and can inhibit the growth of foreign bacteria through the production of bacteriocins and acid production [50].

**Figure 9:**
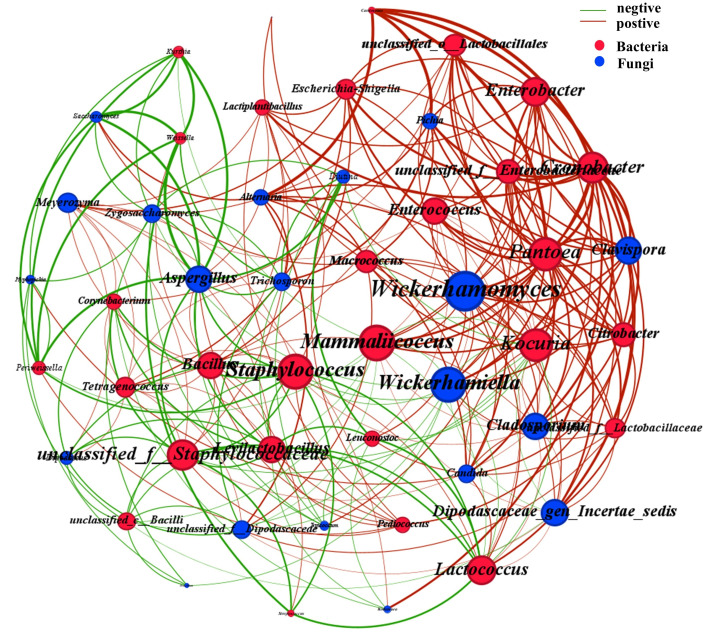
Co-occurrence network of microbial community.

Meanwhile, the interactions among microorganisms are also an important factor in maintaining the stable coexistence of microbial communities. This is crucial for the success and safety of food fermentation as well as for obtaining the desired product characteristics [51]. Network analysis is a type of analysis commonly used to explore the interrelationships among various groups within an ecosystem [52]. A total of 49 nodes were obtained (28 of which were bacterial nodes and 21 were fungal nodes) along with 272 significant correlations (182 positive correlations and 90 negative correlations) (|r| > 0.50, *p* < 0.05) (Figure 9). A positive correlation usually indicates a symbiotic, cooperative or mutually beneficial relationship, while a negative correlation suggests an opposing or competitive relationship [53]. In previous studies, it was found that *Aspergillus* generally demonstrated a negative correlation with other microorganisms during the fermentation process of low-salt soy sauce [54]. This phenomenon was also observed in our research. *Aspergillus* secretes enzyme-like substances, which affect the nutritional network and significantly influence the interactions between different species [55]. On the contrary, some other microorganisms such as *Wickerhamomyces*, *Kocuria* and *Pantoea* show a positive correlation with most microorganisms. Overall, this co-occurrence network demonstrates that positive correlations are much more prevalent than negative correlations, indicating that through the metabolic cooperation of microorganisms, the efficiency of substrate conversion has been enhanced [56]. The present study demonstrates that abiotic factors and microbial interactions significantly influence microbial community structure and succession dynamics, ultimately shaping the fermentation ecosystem of soy sauce.

### 3.6. The Association Between Microorganisms and Specific Volatile Flavor and Taste Substances

**Figure 10:**
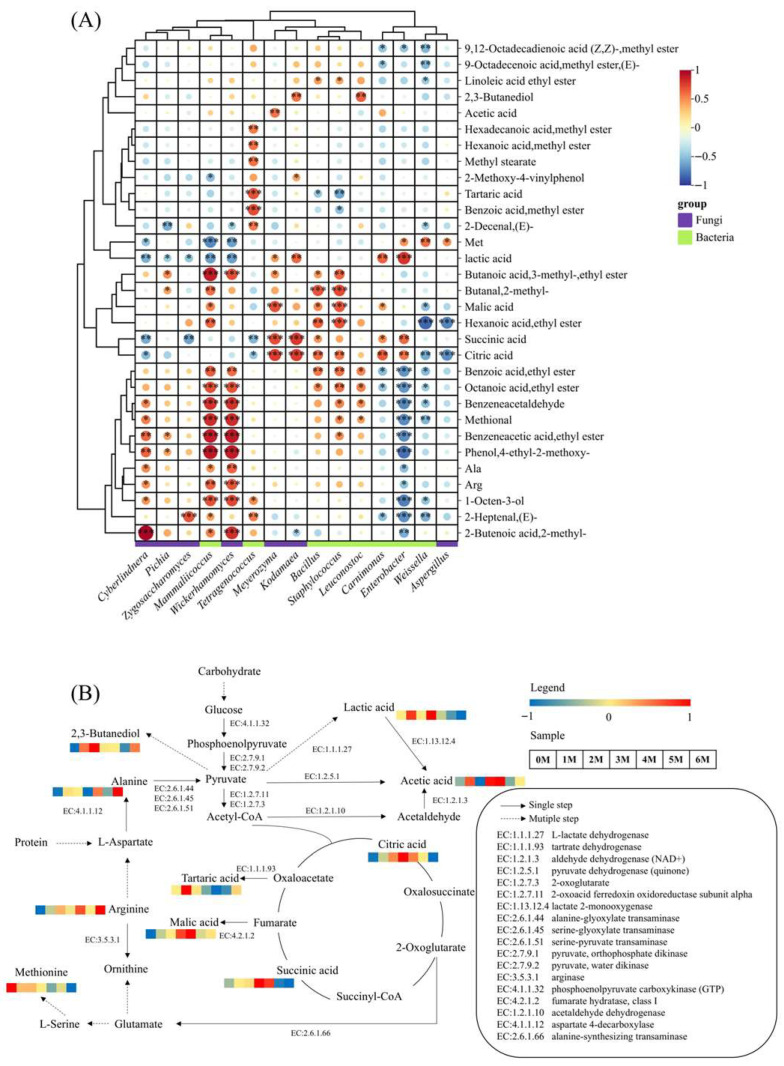
(**A**) Correlation heat map of microorganisms and characteristic flavor compounds in Sichuan-style black soybean soy sauce. *** *p* < 0.001; ** *p* < 0.01; * *p* < 0.05. (**B**) The metabolic pathways of the characteristic metabolites. The heatmap shows the changes in the content of substances in the samples during the fermentation process.

The development of soy sauce flavor and aroma is intrinsically linked to the growth and metabolic activities of microorganisms throughout the fermentation process [57]. To elucidate these relationships, Pearman’s correlation analysis was conducted between 8 high-abundance bacterial genera and 7 fungal genera (relative abundance > 1%), and key aroma-active compounds (ROAV ≥ 1), organic acids, and amino acids (TAV ≥ 1). The results are presented in Figure 10A. The analysis revealed that the vast majority of microorganisms showed positive correlations with flavor compounds. Notably, *Wickerhamomyces* exhibited a broad positive correlation with nearly all aroma compounds (except acids), particularly with key aroma-active substances such as octanoic acid, ethyl ester (r = 0.69), methional (r = 0.83) and Phenol, 4-ethy-2-methoxy- (r = 0.89). Phenol, 4-ethy-2-methoxy-, a key component responsible for soy sauce’s unique aroma, is generated through the specific enzymatic actions of *Wickerhamomyces*. This yeast exclusively possesses both cinnamate decarboxylase and vinylbenzene reductase, which sequentially convert the precursor coumaric acid into 4-vinylguaiacol and then reduce it into Phenol, 4-ethy-2-methoxy- [58]. Previous studies have shown that there is a negative correlation between *Aspergillus* and most flavor compounds [18], and our research also revealed similar results. *Aspergillus* secretes hydrolases to break down the raw materials, promoting the release of flavor substances and nutrients [59], and it is closely related to the formation of free amino acids. By analyzing the correlation between microorganisms and characteristic flavor substances, potential flavor-producing microorganisms can be screened out. Subsequently, this was verified through fermentation experiments and finally applied to industrial production.

Based on the PICRUSt analysis and the KEGG database, a metabolic network of Sichuan-style black soybean soy sauce was constructed (Figure 10B). The carbohydrates in the raw materials were first broken down by amylase and cellulase into fermentable monosaccharides, and then converted into pyruvic acid through the glycolysis pathway [59]. Pyruvic acid is an important intermediate that can serve as a precursor substance for the subsequent biosynthesis of flavor compounds [60]. Pyruvic acid is converted into lactic acid through the action of L-lactate dehydrogenase (EC:1.1.1.27). It is also produced from pyruvate through pyruvate dehydrogenase (quinone) (EC:1.2.5.1) as acetic acid. Pyruvate can also be converted into acetyl-CoA through the action of 2-oxoglutarate/2-oxoacid ferredoxin oxidoreductase subunit alpha (EC: 1.2.7.3 1.2.7.11), and then enter the TCA cycle. In the TCA cycle, citric acid and succinic acid are produced. Simultaneously, Fumarate and Oxaloacetate are generated. These two compounds are then converted into malic acid and tartaric acid, respectively, under the action of fumarate hydratase, class I (EC:4.2.1.2) and tartrate dehydrogenase (EC: 1.1.1.93). Meanwhile, the umami taste-causing glutamate can be produced through the intermediate product 2-Oxoglutarate of the TCA cycle, under the action of alanine-synthesizing transaminase (EC:2.6.1.66). In amino acid metabolism, aspartic acid is an important precursor substance for flavor compounds [60]. The key taste-active amino acid, Alanine, in soy sauce is generated from L-Aspartate under the action of aspartate 4-decarboxylase (EC:4.1.1.12). Amino acids are also precursors of aldehydes, and the main aldehydes in soy sauce, such as phenylacetaldehyde, 3-methylbutylaldehyde and 2-methylbutylaldehyde, are formed from amino acids [10]. Furthermore, pyruvic acid can be converted into 2,3-butanediol flavor metabolites through the biosynthesis pathways of amino acids such as valine and leucine [61].

## 4. Conclusions

This study offers comprehensive insights into the dynamic interplay between physicochemical parameters, flavor development, and microbial succession during the fermentation of Sichuan-style black soybean soy sauce. The fermentation process was characterized by a clear microbial succession that underpinned flavor development: initial proteolysis by *Aspergillus* and *Weissella* provided essential precursors, while the subsequent dominance of *Tetragenococcus* and aroma-producing yeasts drove the synthesis of key flavor compounds. This microbial succession facilitated the synthesis of key flavor compounds, with *Tetragenococcus* associated with ester formation and *Wickerhamomyces* playing a critical role in the production of aroma-active compounds, including methional and 4-ethyl-2-methoxyphenol. These findings provide valuable mechanistic insights into the microecological processes driving flavor formation in traditional Sichuan-style soy sauce. Moreover, the research enhances the scientific understanding of regional fermentation systems and offers a theoretical foundation for microbial management and optimization of production conditions, aimed at improving the quality, consistency, and distinct sensory characteristics of Sichuan-style black soybean soy sauce.

## Figures and Tables

**Table 1:** TAVs of organic acids and free amino acids of Sichuan-style black soybean soy sauce during six-month fermentation.

Compounds	Taste Attributes	Threshold (mg/mL) ^1^	Content (mg/mL)							TAV ^2^						
			0 M	1 M	2 M	3 M	4 M	5 M	6 M	0 M	1 M	2 M	3 M	4 M	5 M	6 M
Oxalic acid	Sour	0.504	0.07 ± 0.00	0.14 ± 0.03	0.19 ± 0.05	0.07 ± 0.00	0.03 ± 0.02	0.03 ± 0.01	0.02 ± 0.01	<1	<1	<1	<1	<1	<1	<1
Tartaric acid	Sour	0.3	0.04 ± 0.00	0.60 ± 0.11	0.06 ± 0.01	0.02 ± 0.00	0.01 ± 0.00	0.02 ± 0.01	0.10 ± 0.01	<1	2.01	<1	<1	<1	<1	<1
Malic acid	Sour	0.495	0.07 ± 0.01	0.25 ± 0.05	0.31 ± 0.00	0.63 ± 0.00	0.76 ± 0.21	0.27 ± 0.04	0.32 ± 0.04	<1	<1	<1	1.27	1.53	<1	<1
Lactic acid	Sour	1.26	1.89 ± 0.03	2.97 ± 0.16	1.91 ± 0.06	3.30 ± 0.09	1.60 ± 0.12	1.06 ± 0.06	0.64 ± 0.06	1.50	2.36	1.52	2.62	1.27	<1	<1
Acetic acid	Sour	0.0768	0.10 ± 0.03	0.16 ± 0.05	0.07 ± 0.01	0.17 ± 0.03	0.17 ± 0.02	0.10 ± 0.01	0.13 ± 0.01	1.32	2.04	<1	2.21	2.25	1.26	1.65
Citric acid	Sour	0.45	0.34 ± 0.09	0.61 ± 0.02	0.88 ± 0.01	1.16 ± 0.02	0.91 ± 0.09	0.68 ± 0.03	0.32 ± 0.03	<1	1.35	1.96	2.58	2.01	1.52	<1
Succinic acid	Sour	0.106	0.72 ± 0.00	0.87 ± 0.03	0.88 ± 0.02	1.06 ± 0.02	1.01 ± 0.16	0.51 ± 0.03	0.46 ± 0.03	6.77	8.19	8.29	9.95	9.54	4.85	4.37
Aspartate	Umami	1	0.03 ± 0.01	0.29 ± 0.07	0.26 ± 0.04	0.05 ± 0.02	0.03 ± 0.01	0.17 ± 0.03	0.37 ± 0.03	<1	<1	<1	<1	<1	<1	<1
Glutamine	Umami	0.3	0.13 ± 0.02	0.20 ± 0.04	0.20 ± 0.02	0.14 ± 0.03	0.20 ± 0.10	0.13 ± 0.08	0.19 ± 0.08	<1	<1	<1	<1	<1	<1	<1
Serine	Sweet	1.5	0.09 ± 0.00	0.18 ± 0.04	0.18 ± 0.02	0.15 ± 0.04	0.24 ± 0.10	0.20 ± 0.10	0.34 ± 0.10	<1	<1	<1	<1	<1	<1	<1
Glycine	Sweet	1.3	0.03 ± 0.01	0.05 ± 0.02	0.05 ± 0.01	0.04 ± 0.01	0.05 ± 0.00	-	0.03 ± 0.00	<1	<1	<1	<1	<1	-	<1
Histidine	Bitter	0.9	0.04 ± 0.01	0.04 ± 0.00	0.05 ± 0.00	0.04 ± 0.00	-	-	0.04 ± 0.00	<1	<1	<1	<1	-	-	<1
Arginine	Bitter	0.5	0.95 ± 0.08	1.82 ± 0.42	2.51 ± 0.28	2.05 ± 0.50	3.19 ± 1.60	2.11 ± 1.47	3.74 ± 1.47	1.89	3.64	5.02	4.10	6.39	4.21	7.48
Threonine	Sweet	2.6	0.07 ± 0.02	0.04 ± 0.00	0.03 ± 0.01	0.03 ± 0.00	0.06 ± 0.00	0	0.05 ± 0.00	<1	<1	<1	<1	<1	-	<1
Alanine	Sweet	0.6	3.50 ± 0.25	4.14 ± 0.97	4.00 ± 0.46	3.47 ± 0.84	5.23 ± 2.60	3.73 ± 2.57	6.53 ± 2.57	5.83	6.90	6.67	5.79	8.71	6.21	10.89
Proline	Sweet	3	0.03 ± 0.00	0.06 ± 0.02	0.04 ± 0.00	0.04 ± 0.01	0.06 ± 0.00	0.05 ± 0.00	0.05 ± 0.00	<1	<1	<1	<1	<1	<1	<1
Valine	Bitter	0.4	0.03 ± 0.00	0.04 ± 0.02	0.04 ± 0.00	0.03 ± 0.01	0.08 ± 0.04	0.06 ± 0.04	0.05 ± 0.04	<1	<1	<1	<1	<1	<1	<1
Methionine	Bitter	0.3	0.76 ± 0.03	0.64 ± 0.04	0.65 ± 0.06	0.61 ± 0.02	0.57 ± 0.06	0.60 ± 0.06	0.53 ± 0.06	2.53	2.14	2.15	2.04	1.90	2.01	1.76
Cysteine	Tasteless	/	0.06 ± 0.02	0.58 ± 0.19	0.61 ± 0.16	0.51 ± 0.13	0.24 ± 0.05	0.76 ± 0.55	1.55 ± 0.55	/	/	/	/	/	/	/
Isoleucine	Bitter	0.9	0.03 ± 0.00	0.03 ± 0.01	0.04 ± 0.00	0.03 ± 0.00	0.06 ± 0.02	0.09 ± 0.08	0.15 ± 0.08	<1	<1	<1	<1	<1	<1	<1
Leucine	Bitter	1.9	0.11 ± 0.07	0.30 ± 0.09	0.31 ± 0.03	0.23 ± 0.05	0.30 ± 0.15	0.24 ± 0.17	0.42 ± 0.17	<1	<1	<1	<1	<1	<1	<1
Phenylalanine	Bitter	0.2	0.05 ± 0.00	0.05 ± 0.00	1.02 ± 0.11	0.84 ± 0.19	0.05 ± 0.01	0.05 ± 0.01	0.06 ± 0.01	<1	<1	<1	<1	<1	<1	<1
Tryptophan	Bitter	/	0.47 ± 0.09	1.05 ± 0.31	1.44 ± 1.23	1.55 ± 0.38	1.44 ± 0.72	0.94 ± 0.65	1.91 ± 0.65	/	/	/	/	/	/	/

“/”, not available. “-”, not detected. ^1^ Threshold concentrations were taken from [26]. ^2^ TAV is calculated as the ratio of the content and taste threshold. 0–6 M, samples that were fermented from month 0 to month 6.

**Table 2:** The aroma-active compounds (ROAV > 1) of Sichuan-style black soybean soy sauce during six-month fermentation.

Compounds	Odor Threshold (μg/L) ^1^	Odor Description ^2^	ROAV ^3^						
			0 M	1 M	2 M	3 M	4 M	5 M	6 M
Phenol, 4-ethyl-2-methoxy-	89.3	spicy and clove-like with medicinal	<1	4.70	13.11	2.75	29.78	19.15	36.36
2-Methoxy-4-vinylphenol	12.02	dry, woody, fresh	21.63	181.11	111.63	3.55	25.28	15.63	14.10
Butanal, 2-methyl-	16	musty, chocolate, nutty	<1	<1	<1	<1	26.76	<1	<1
2-Heptenal, (E)-	40	green, fatty, oily, fruity	<1	1.21	1.04	<1	1.35	2.60	1.34
Methional	0.45	musty, tomato, potato	4.77	53.20	79.61	37.04	114.66	66.67	119.05
Benzeneacetaldehyde	235.3	honey, floral rose, sweet	<1	<1	2.32	<1	2.89	1.80	3.11
2-Decenal, (E)-	10	waxy, fatty, earthy	<1	1.48	1.15	<1	<1	1.28	<1
2-Butenoic acid, 2-methyl-	5.8	sour	<1	<1	<1	<1	<1	<1	1.32
1-Octen-3-ol	1.5	mushroom, earthy	40.68	125.87	74.01	68.01	144.23	101.95	149.35
2,3-Butanediol	100	fruity, creamy, oily	<1	<1	1.17	<1	<1	<1	<1
Butanoic acid, 3-methyl-, ethyl ester	6.89	sweet, diffusive, estry	<1	<1	<1	3.12	8.85	1.82	4.81
Hexanoic acid, methyl ester	14	ethereal, fruity, pineapple,	<1	5.94	<1	<1	2.74	1.86	<1
Hexanoic acid, ethyl ester	5	fruity, winey, waxy	<1	17.52	30.87	23.47	41.98	38.04	23.44
Octanoic acid, ethyl ester	12.9	waxy, sweet, musty	<1	16.33	73.03	<1	80.26	45.91	76.13
Benzoic acid, methyl ester	73	floral, fruity	<1	7.56	<1	<1	1.59	1.82	0.73
Benzoic acid, ethyl ester	1430	sweet, wintergreen, fruity	<1	<1	1.75	<1	1.87	1.38	1.58
Benzeneacetic acid, ethyl ester	407	honey	<1	<1	<1	<1	1.54	<1	1.85
Hexadecanoic acid, methyl ester	2	oily, waxy, fatty	234.31	1648.23	448.54	95.39	836.49	716.20	207.97
Methyl stearate	3	oily, waxy	3.26	33.35	11.77	1.21	13.23	18.14	2.38
9-Octadecenoic acid, methyl ester, (E)-	40	oily	1.64	11.02	9.95	1.02	8.88	8.80	2.48
9,12-Octadecadienoic acid (Z,Z)-, methyl ester	35	oily, fatty, woody	4.45	28.53	15.81	3.39	27.54	23.29	9.20
Linoleic acid ethyl ester	4.5	fatty, fruity, oily	1.44	24.19	48.65	1.40	37.22	22.48	25.40

^1^ Odor threshold of each compound was obtained from references [8,15,33]. ^2^ Odor description of each compound was obtained from http://www.perflavory.com/(accessed on 20 August 2025). ^3^ ROAV is calculated as the ratio of the content and odor threshold. 0–6 M, samples that were fermented from month 0 to month 6.

## Data Availability

The original contributions presented in the study are included in the article/Supplementary Material, further inquiries can be directed to the corresponding author.

## Supplementary Materials

The following supporting information can be downloaded at: https://www.mdpi.com/article/10.3390/foods14234049/s1, Table S1: Standard Curves for Organic Acids and Amino Acids. Table S2: The concentration of volatile flavor compounds during the fermentation of Sichuan-style black soybean soy sauce.
