# Supplementary material for: Insights into Physicochemical Characteristics, Flavor Development, and Microbial Succession During the Natural Fermentation of Sichuan-Style Black Soybean Soy Sauce
Source: Foods. 2025 Nov 26;14(23):4049. doi: 10.3390/foods14234049 (PMC12692206; doi:10.3390/foods14234049)
Supplement: Supplementary file 1 [file foods-14-04049-s001.zip › foods-3989939-supplementary.pdf]

Insights into Physicochemical Characteristics, Flavor Development, and Microbial Succession during the Natural Fermentation of Sichuan-Style Black Soybean Soy Sauce

Yutian Xie [1], Shenglan Liao [1], Youming Li [1], Xianbin Wang [4], Yunhao Lu [1,2], Qixu Fu [3], Qiang He [1], Yuanlong Chi [1,2] * and Zhenghong Xu [1,2]

[1] Innovation Center for Advanced Brewing Science and Technology, College of Biomass Science and Engineering, Sichuan University, Chengdu, 610065, China
[2] Key Laboratory of Monitoring and Assessment on Novel Food Raw Materials, State Administration for Market Regulation, Sichuan University, Chengdu, 610065, China
[3] Luzhou Laojiao Group Co., Ltd., Luzhou, 646000, China
[4] Yuanjingda Food Co., Ltd, Luzhou, 646000, China
* Correspondence: chiyl@scu.edu.cn

Table S1. Standard Curves for Organic Acids and Amino Acids

Coupounds	Standard curves	R^2
Oxalic acid	y=10840x+326.2	0.9994
Tartaric acid	y=1931.3x+79.265	0.9991
Malic acid	y=981.37x-1.5839	1
Lactic acid	y=539.06x-0.05	1
Acetic acid	y=705.3x-0.9157	1
Citric acid	y=7356.71x+137.24	0.9981
Succinic acid	y=2110.4x-50.661	0.9995
Aspartate	y=236.19x+0.5314	0.9959
Glutamine	y=289.55x+1.0075	0.9996
Serine	y=338.59x-9.4659	0.9954
Glycine	y=309.48x+24.215	0.9983
Histidine	y=346.07x-2.6034	0.999
Arginine	y=314.1x-1.58	0.9949
Threonine	y=264.53x-3.5274	0.9975
Alanine	y=249.7x+1.0857	0.9988
Proline	y=327.69x-5.6763	0.9961
Valine	y=217.28x+1.58	0.9976
Methionine	y=328.92x-1.1725	0.9975
Cysteine	y=346.92x+1.8543	0.9986
Isoleucine	y=179.68x+0.03	0.9978
Leucine	y=311.04x+1.76	0.9994
Phenylalanine	y=418.83x-18.84	0.9912
Tryptophan	y=342.33x-6.1368	0.9951

Table S2. The concentration of volatile flavor compounds during the fermentation of Sichuan-style black soybean soy sauce.

Ret.Time	Name	CAS	Concentration (μg/L)						
			0M	1M	2M	3M	4M	5M	6M
	Esters								
2.815	Ethyl Acetate	141 - 78 - 6	-	34.16±11.65	287.56±89.61	806.10±70.70	619.09±37.07	845.06±104.60	826.87±108.25
5.531	Butanoic acid, 2-methyl-, ethyl ester	7452 - 79 - 1	-	-	-	94.17±126.78	71.74±3.75	37.78±8.16	55.99±5.22
5.881	Butanoic acid, 3-methyl-, ethyl ester	108 - 64 - 5	-	-	-	21.46±1.32	60.97±5.17	12.54±2.07	33.11±4.40
7.177	1-Butanol, 3-methyl-, acetate	123 - 92 - 2	-	-	67.38±8.07	-	69.32±11.61	32.70±5.23	56.97±8.64
8.197	2-Butenoic acid, ethyl ester, (E)-	623 - 70 - 1	-	-	-	9.61±0.65	-	-	-
8.979	Hexanoic acid, methyl ester	106 - 70 - 7	-	83.15±6.53	-	-	38.37±5.98	25.99±3.68	-
10.329	Hexanoic acid, ethyl ester	123 - 66 - 0	-	87.60±11.45	154.35±19.15	117.34±8.39	209.91±21.55	190.18±18.66	117.22±25.50
11.758	Heptanoic acid, methyl ester	106 - 73 - 0	-	44.29±2.96	-	14.88±1.02	-	17.70±1.68	-
13.05	Heptanoic acid, ethyl ester	106 - 30 - 9	-	-	-	-	25.12±5.93	-	-
13.099	Propanoic acid, 2-hydroxy-, ethyl ester	97 - 64 - 3	-	86.64±15.43	-	37.28±5.06	-	-	480.98±103.19

14.726	Octanoic acid, methyl ester	111 - 11 - 5	-	239.39±18.47	91.31±30.69	50.38±4.82	303.13±35.07	175.99±4.17	101.77±16.95
16.509	Octanoic acid, ethyl ester	106 - 32 - 1	-	210.63±38.01	941.98±156.51	-	1035.41±157.84	592.18±30.85	982.03±234.41
19.104	Nonanoic acid, methyl ester	1731 - 84 - 6	14.29±1.93	72.62±10.09	43.24±14.06	45.24±8.04	60.33±33.23	40.59±8.98	11.37±1.63
21.015	Nonanoic acid, ethyl ester	123 - 29 - 5	-	32.36±3.54	156.09±10.29	50.79±10.35	82.58±10.27	100.03±17.47	93.97±14.37
21.291	3-Nonenoic acid, methyl ester	13481 - 87 - 3	-	1.65±0.15	-	-	-	-	-
21.74	3-(Methylthio)propanoic acid ethyl ester	13327 - 56 - 5	-	-	-	-	-	31.73±5.87	-
23.076	Decanoic acid, methyl ester	110 - 42 - 9	3.67±0.27	80.85±13.77	95.02±23.59	7.29±2.15	191.73±40.32	81.21±7.77	49.67±9.93
23.135	Butanoic acid, 4-hydroxy-	591 - 81 - 1	-	-	-	14.57±2.17	-	-	-
23.15	Butyrolactone	96 - 48 - 0	1.00±0.18	-	-	-	-	-	-
23.388	Benzoic acid, methyl ester	93 - 58 - 3	29.88±2.43	551.62±87.95	15.25±5.46	-	115.78±12.56	132.92±8.41	53.40±2.22
23.391	Benzoyl isothiocyanate	532 - 55 - 8	-	-	-	11.82±2.83	-	-	-
24.154	Butanedioic acid, ethyl methyl ester	627 - 73 - 6	-	47.60±3.34	-	40.98±9.41	388.60±22.63	187.08±7.82	232.38±41.29
24.526	Decanoic acid, ethyl ester	110 - 38 - 3	-	51.19±1.24	468.75±164.45	29.51±9.07	391.26±32.08	181.72±25.20	201.76±44.19
24.859	Benzeneacetic acid, .alpha.-oxo-,	15206 - 55	-	-	-	478.43±195.62	-	-	-

	methyl ester	- 0							
24.869	Benzoic acid, ethyl ester	93 - 89 - 0	17.04±0.87	714.73±194.47	2503.76±625.67	-	2668.69±290.15	1968.61±170.75	2259.57±459.22
24.884	Octanoic acid, 3-methylbutyl ester	2035 - 99 - 6	-	-	12.39±7.36	-	-	-	-
25.045	Methyl formate	107 - 31 - 3	-	-	-	25.72±4.27	-	-	-
25.289	4-Decenoic acid, ethyl ester, (Z)-	7367 - 84 - 2	-	-	57.26±27.04	-	79.59±2.67	28.50±6.79	35.49±3.60
25.439	Butanedioic acid, diethyl ester	123 - 25 - 1	-	-	447.74±78.96	35.99±3.56	549.59±11.82	305.03±19.42	535.45±89.66
27.415	Undecanoic acid, ethyl ester	627 - 90 - 7	-	7.57±1.03	53.39±21.56	4.68±1.52	55.13±6.78	5.16±1.02	5.15±0.88
27.483	Benzeneacetic acid, methyl ester	101 - 41 - 7	1.20±0.59	-	-	-	17.48±3.24	8.73±0.28	-
28.202	Benzeneacetic acid, ethyl ester	101 - 97 - 3	1.21±0.30	51.67±5.54	360.41±85.03	95.57±20.56	625.53±79.40	313.03±33.03	753.33±142.10
28.691	Ethyl nicotinate	614 - 18 - 6	-	4.19±0.78	30.89±11.96	4.98±1.71	48.58±3.76	12.46±2.32	9.68±1.25
28.887	Acetic acid, 2-phenylethyl ester	103 - 45 - 7	-	-	139.72±48.31	14.03±5.36	-	160.81±13.17	75.94±15.23
28.917	Dodecanoic acid, methyl ester	111 - 82 - 0	3.76±0.75	77.30±12.53	-	-	181.24±33.93	-	-
29.614	Benzenepropanoic acid, methyl ester	103 - 25 - 3	-	16.67±1.44	-	-	26.28±4.96	12.32±0.87	-
29.946	Dodecanoic acid, ethyl ester	106 - 33 -	-	38.31±11.22	425.38±179.70	15.40±3.09	354.37±41.12	171.27±29.13	161.63±28.36

		2							
30.569	Benzenepropanoic acid, ethyl ester	2021 - 28 - 5	-	-	23.45±8.20	-	-	-	-
31.322	Ethyl tridecanoate	28267 - 29 - 0	-	-	-	-	75.15±8.74	45.31±6.47	45.00±10.84
33.136	Methyl tetradecanoate	124 - 10 - 7	-	-	-	-	5.86±0.50	2.39±0.64	-
34.413	2(3H)-Furanone, dihydro-5-pentyl-	104 - 61 - 0	-	7.28±2.09	8.24±2.56	-	8.88±0.91	25.67±4.32	14.62±2.45
35.253	9-Octadecenoic acid (Z)-, methyl ester	112 - 62 - 9	-	4.10±0.76	-	-	-	-	-
36.286	Tetradecanoic acid, ethyl ester	124 - 06 - 1	-	46.39±19.47	412.34±197.41	34.86±3.61	368.17±28.74	233.99±56.67	230.09±48.98
37.076	Tetradecanoic acid, 12-methyl-, methyl ester	5129 - 66 - 8	-	-	-	-	16.70±1.69	-	-
37.088	Eicosanoic acid, methyl ester	1120 - 28 - 1	-	11.10±2.66	-	-	-	9.57±1.00	-
37.946	Pentadecanoic acid, methyl ester	7132 - 64 - 1	3.91±1.44	32.33±5.49	7.08±2.14	2.00±0.24	19.12±3.70	15.77±2.22	1.97±0.24
38.094	Eicosanoic acid, ethyl ester	18281 - 05 - 5	-	-	-	4.62±0.38	22.77±2.40	32.01±8.44	22.80±4.67
40.314	Hexadecanoic acid, methyl ester	112 - 39 - 0	468.62±105.11	3296.46±576.26	897.07±260.92	190.78±10.54	1672.98±294.69	1432.40±593.05	415.94±68.63
40.9	9-Hexadecenoic acid, methyl ester, (Z)-	1120 - 25 - 8	3.02±0.84	19.66±3.57	8.94±3.19	1.98±0.42	15.58±3.21	11.47±2.38	3.74±1.35

41.424	Hexadecanoic acid, ethyl ester	628 - 97 - 7	44.08±23.28	1787.98±483.18	7280.86±3933.04	503.89±37.93	4795.41±471.00	3276.25±1077.06	3143.23±676.29
43.247	Heptadecanoic acid, methyl ester	1731 - 92 - 6	0.84±0.33	10.99±2.46	-	-	-	4.82±1.65	-
44.97	Ethyl 9-hexadecenoate	54546 - 22 - 4	-	15.91±3.27	81.09±45.69	4.69±1.82	53.71±5.10	27.94±6.50	4.11±1.37
46.011	Methyl stearate	112 - 61 - 8	9.77±3.80	100.05±21.19	35.32±9.71	3.63±0.48	39.70±10.29	54.41±18.44	7.13±0.51
46.342	9-Octadecenoic acid, methyl ester, (E)-	1937 - 62 - 8	65.49±17.27	440.80±151.05	397.86±102.74	40.84±4.31	355.37±78.65	352.13±85.64	99.24±8.88
46.481	11-Octadecenoic acid, methyl ester	52380 - 33 - 3	8.38±1.53	58.75±11.34	-	-	27.95±6.22	22.03±5.87	5.70±0.56
46.738	Octadecanoic acid, ethyl ester	111 - 61 - 5	4.23±1.96	59.88±10.63	205.27±122.56	10.42±0.33	122.84±26.14	65.26±8.17	69.85±17.36
47.023	Ethyl Oleate	111 - 62 - 6	13.21±4.18	353.20±79.52	1682.37±982.45	109.15±6.95	993.35±177.19	517.24±31.52	642.25±155.46
47.155	9,12-Octadecadienoic acid (Z,Z)-, methyl ester	112 - 63 - 0	155.67±20.93	998.62±235.29	553.36±205.58	118.52±25.27	964.05±171.02	815.30±135.82	321.96±27.93
48.138	9,12,15-Octadecatrienoic acid, methyl ester, (Z,Z,Z)-	301 - 00 - 8	16.60±7.26	4.83±1.87	28.34±15.39	3.59±0.05	41.30±6.93	19.48±8.00	25.11±1.68
48.311	Benzoic acid, 4-hydroxy-3-methoxy-, methyl ester	3943 - 74 - 6	-	10.03±3.39	-	-	4.38±1.13	2.79±0.41	-
48.622	9,12,15-Octadecatrienoic acid, ethyl ester, (Z,Z,Z)-	1191 - 41 - 9	-	60.96±21.07	372.59±140.46	24.14±2.66	265.23±45.66	155.36±9.01	140.27±33.22
48.666	12,15-Octadecadienoic acid, methyl	57156 - 97	-	28.62±3.66	-	-	-	-	-

	ester	- 5							
48.719	Benzoic acid, 4-hydroxy-3-methoxy-, ethyl ester	617 - 05 - 0	-	-	-	-	4.60±1.24	1.86±0.33	8.71±1.87
49.271	9,11-Octadecadienoic acid, methyl ester, (E,E)-	13038 - 47 - 6	7.87±0.14	33.43±4.18	-	4.28±1.19	36.77±4.46	24.00±7.39	8.60±0.34
49.681	Linoleic acid ethyl ester	544 - 35 - 4	6.46±2.87	108.86±50.18	218.94±171.69	6.30±1.47	167.48±35.98	101.18±16.86	114.31±14.51
	Alcohols								
3.398	Ethanol	64 - 17 - 5	45.69±5.05	2195.09±398.48	15416.07±2490.37	7669.14±4492.35	7762.07±481.86	12415.89±360.78	14502.23±2221.63
6.761	1-Propanol, 2-methyl-	78 - 83 - 1	-	24.46±2.62	188.60±18.72	30.45±6.98	163.52±18.26	84.01±5.81	115.76±29.19
7.882	1-Butanol	71 - 36 - 3	-	-	119.46±13.75	15.94±3.57	79.60±7.74	23.18±1.66	24.16±5.56
9.526	1-Butanol, 3-methyl-	123 - 51 - 3	-	324.48±23.06	1868.03±195.87	408.34±45.42	1110.09±62.61	617.45±40.87	799.32±171.77
10.638	1-Pentanol	71 - 41 - 0	2.57±0.15	-	-	-	-	-	-
11.371	Acetoin	513 - 86 - 0	2.30±0.28	19.34±2.78	10.47±0.28	7.13±1.10	25.16±5.04	15.20±1.99	17.45±4.35
12.393	Propylene Glycol	1569 - 01 - 3	-	11.24±2.48	-	-	-	-	-
13.352	1-Decanol, 2-hexyl-	2425 - 77 - 6	-	1.87±0.46	-	-	-	-	-
13.475	1-Hexanol	111 - 27 - 3	-	3.17±0.26	-	3.60±2.16	-	7.22±2.11	6.14±0.44
14.849	3-Octanol	589 - 98 - 0	2.80±0.15	1.40±0.29	49.05±66.98	-	11.87±0.71	13.62±0.12	10.35±0.81
17.113	1-Octen-3-ol	3391 - 86 -	61.02±6.37	188.81±18.13	111.02±11.16	102.02±18.03	216.34±20.59	152.92±3.31	224.03±27.65

		4							
18.99	1-Hexanol, 2-ethyl-	104 - 76 - 7	1.08±0.23	-	-	11.05±2.84	-	4.44±0.83	8.40±0.13
19.785	2-Hepten-1-ol, (E)-	33467 - 76 - 4	1.18±0.22	4.45±1.05	-	-	-	-	-
20.666	2,3-Butanediol	513 - 85 - 9	22.87±0.98	86.64±14.20	117.45±25.85	54.75±2.96	55.47±6.27	30.52±2.16	84.37±18.09
22.127	Ethanol, 2-methoxy-	109 - 86 - 4	-	-	-	45.84±7.96	-	-	-
23.56	Cyclooctyl alcohol	696 - 71 - 9	-	3.91±0.94	-	-	-	-	-
23.648	trans-2-Undecen-1-ol	75039 - 84 - 8	-	15.73±1.43	-	-	-	-	-
26.278	1-Propanol, 3-(methylthio)-	505 - 10 - 2	-	34.51±1.79	90.73±12.53	82.51±4.34	140.31±17.52	87.01±7.01	145.08±14.06
30.246	Benzyl alcohol	100 - 51 - 6	-	-	19.33±5.28	7.29±1.78	-	-	-
31.09	Phenylethyl Alcohol	22258	7.36±1.58	258.44±59.30	2416.35±362.37	271.99±59.35	1799.37±259.01	1111.28±111.90	1186.40±113.06
33.26	.beta.-Ethylphenethyl alcohol	2035 - 94 - 1	-	-	-	5.73±0.67	7.23±1.79	4.00±0.81	3.96±1.15
	Acids								
16.536	Acetic acid	64 - 19 - 7	12.18±2.45	-	-	221.43±40.45	-	-	-
23.73	Butanoic acid	107 - 92 - 6	-	-	-	-	3.48±1.01	-	-
25.052	Pentanoic acid, 3-methyl-	105 - 43 -	9.68±0.98	55.88±10.40	-	-	-	40.55±3.63	-

		1							
29.545	2-Butenoic acid, 2-methyl-	13201 - 46 - 2	-	-	-	-	-	-	7.66±0.92
29.612	Hexanoic acid	142 - 62 - 1	-	-	-	-	-	-	13.90±2.01
43.06	9,12-Octadecadienoic acid (Z,Z)-	60 - 33 - 3	-	-	-	-	4.26±0.49	2.27±0.07	-
45.297	Benzoic acid	65 - 85 - 0	-	10.46±2.96	-	-	11.38±1.63	7.13±2.35	6.33±1.89
52.388	n-Hexadecanoic acid	21096	1.49±0.34	15.51±3.21	-	2.65±1.00	32.15±4.99	18.86±4.41	15.49±3.23
	Aldehydes								
3.021	Butanal, 2-methyl-	96 - 17 - 3	-	-	-	-	428.19±46.86	-	-
12.513	2-Heptenal, (E)-	18829 - 55 - 5	-	48.41±6.24	41.57±15.68	-	53.87±11.25	104.06±13.05	53.79±8.37
16.751	Methional	3268 - 49 - 3	2.15±0.45	23.94±3.78	35.83±4.49	16.67±1.86	51.60±1.75	30.00±3.33	53.57±3.39
17.188	Furfural	35796	-	-	123.66±18.50	68.32±19.14	103.99±17.66	106.06±7.49	326.54±59.42
19.216	Decanal	112 - 31 - 2	2.93±0.58	21.78±3.61	12.11±4.57	-	17.10±2.34	-	11.41±2.24
19.647	Benzaldehyde	100 - 52 - 7	12.98±1.30	103.12±21.45	423.89±90.59	72.53±14.93	383.88±53.15	249.15±18.25	333.89±40.62
20.463	2-Nonenal, (E)-	18829 - 56 - 6	2.00±0.45	17.30±2.01	11.78±1.93	-	-	10.44±1.97	32.39±15.85
23.932	Benzeneacetaldehyde	122 - 78 - 1	23.84±3.93	227.75±54.20	545.45±86.80	145.66±31.42	680.58±39.01	422.93±27.30	730.82±36.06
24.257	2-Decenal, (E)-	3913 - 81 - 3	-	14.76±2.30	11.50±2.92	-	-	12.78±2.47	-

31.514	Benzeneacetaldehyde, .alpha.-ethylidene-	4411 - 89 - 6	-	24.19±6.97	86.93±28.18	47.46±10.12	110.18±19.67	41.40±2.90	86.57±11.27
32.442	2H-Pyran-2-carboxaldehyde, 3,4-dihydro-	100 - 73 - 2	-	-	-	-	10.01±2.73	-	-
	Phenols								
29.824	Phenol, 2-methoxy-	32994	-	4.15±1.21	12.00±2.41	4.03±1.35	14.92±1.68	6.20±0.83	14.94±2.18
32.266	Maltol	118 - 71 - 8	2.82±0.04	22.37±5.82	37.48±9.00	-	78.40±23.48	37.89±8.76	63.11±5.69
34.864	Phenol, 4-ethyl-2-methoxy-	89 - 84 - 9	-	537.32±109.78	1170.47±357.64	245.80±46.59	2659.26±457.94	1709.86±247.49	3247.14±459.05
38.829	Phenol, 4-ethyl-	123 - 07 - 9	14.92±2.22	155.30±31.28	406.30±139.62	87.73±21.49	615.25±100.19	339.08±52.02	436.35±54.83
39.179	2-Methoxy-4-vinylphenol	7786 - 61 - 0	260.04±185.34	2176.96±449.33	1341.83±592.69	42.71±12.55	303.81±21.71	187.90±12.53	169.45±24.84
	Ketones								
12.028	1-Hepten-3-one	2918 - 13 - 0	4.95±0.32	19.10±1.70	47.95±15.42	18.62±4.90	37.03±3.37	67.04±7.24	42.50±6.13
12.744	2,3-Octanedione	585 - 25 - 1	-	7.52±0.94	13.79±2.44	-	11.54±2.05	19.16±2.38	16.57±3.21
12.982	5-Hepten-2-one, 6-methyl-	110 - 93 - 0	1.72±0.54	-	-	-	-	-	-
25.938	4-Methyl-5H-furan-2-one	6124 - 79 - 4	-	-	15.11±1.27	-	11.86±1.13	-	-
29.98	5,9-Undecadien-2-one, 6,10-dimethyl-	689 - 67 - 8	1.01±0.49	-	-	-	-	-	-
33.62	Dehydromevalonic lactone	2381 - 87 -	-	-	-	2.23±0.32	-	-	-

		5							
	Furans								
29.706	Furan, 3-phenyl-	13679 - 41 - 9	-	-	22.09±6.76	-	14.44±1.91	-	10.46±1.69
44.541	Benzofuran, 2,3-dihydro-	496 - 16 - 2	32.54±2.51	247.40±95.19	184.71±87.93	3.69±0.56	24.25±5.12	18.72±4.81	12.07±2.81
	Pyrazines								
12.645	Pyrazine, 2,6-dimethyl-	108 - 50 - 9	-	-	-	26.31±4.42	-	-	-
	Others								
6.44	Tridecane	629 - 50 - 5	-	25.81±4.22	-	41.40±3.58	32.86±1.16	-	44.04±13.89
8.95	Pyridine	110 - 86 - 1	-	-	-	34.56±6.28	-	-	-
10.689	1,3,5,7-Cyclooctatetraene	629 - 20 - 9	-	-	192.91±27.46	-	134.10±10.94	-	58.92±26.16
15.471	Tetradecane	629 - 59 - 4	0.83±0.24	-	31.15±11.06	9.40±4.36	32.79±8.51	9.15±0.40	19.51±2.95
16.175	Benzene, 1,3-bis(1,1-dimethylethyl)-	1014 - 60 - 4	-	-	20.30±4.78	11.71±0.87	-	-	17.27±0.41
18.718	1-Hexene, 3,5,5-trimethyl-	4316 - 65 - 8	1.36±0.10	-	-	-	-	-	-
22.653	Cyclohexane, 1-ethenyl-1-methyl-2,4-bis(1-methylethenyl)-, [1S-(1.alpha.,2.beta.,4.beta.)]-	515 - 13 - 9	-	12.29±2.05	47.02±14.30	15.26±2.04	37.39±4.49	8.83±3.13	67.48±9.67

24.421	6-Methyl-1,2,3,5,8,8a-hexahydronaphthalene	107914 - 86 - 3	-	7.50±1.54	-	-	-	-	-
24.59	Azulene, 1,2,3,3a,4,5,6,7-octahydro-1,4-dimethyl-7-(1-methylethenyl)-, [1R-(1.alpha.,3a.beta.,4.alpha.,7.beta.)]-	22567 - 17 - 5	-	-	-	-	-	-	14.60±1.37
27.526	Naphthalene, 1,2,3,4,4a,5,6,8a-octahydro-7-methyl-4-methylene-1-(1-methylethyl)-, (1.alpha.,4a.beta.,8a.alpha.)-	39029 - 41 - 9	-	25.00±1.80	53.99±17.73	14.06±3.06	32.58±4.44	11.93±0.88	166.82±28.59
29.276	trans-Calamenene	73209 - 42 - 4	-	2.18±0.30	5.80±1.83	2.60±0.60	-	-	14.59±2.57
32.672	Ethanone, 1-(1H-pyrrol-2-yl)-	1072 - 83 - 9	-	-	45.38±16.29	24.32±5.71	41.32±6.35	14.71±3.12	48.85±5.60
35.913	3-Pentanone, 2,2,4,4-tetramethyl-	123 - 62 - 6	-	7.78±5.47	72.59±24.73	27.03±4.06	345.88±104.55	322.69±79.34	226.37±41.94

Note: “-”, not detect. 0-6M, samples which fermentation from month 0 to month 6.
